# Loss of *Pten* Causes Tumor Initiation Following Differentiation of Murine Pluripotent Stem Cells Due to Failed Repression of *Nanog*

**DOI:** 10.1371/journal.pone.0016478

**Published:** 2011-01-27

**Authors:** Anne G. Lindgren, Kyle Natsuhara, E. Tian, John J. Vincent, Xinmin Li, Jing Jiao, Hong Wu, Utpal Banerjee, Amander T. Clark

**Affiliations:** 1 Department of Molecular Cell and Developmental Biology, University of California Los Angeles, Los Angeles, California, United States of America; 2 Molecular Biology Institute, University of California Los Angeles, Los Angeles, California, United States of America; 3 Jonsson Comprehensive Cancer Center, University of California Los Angeles, Los Angeles, California, United States of America; 4 Department of Pathology and Laboratory Medicine, University of California Los Angeles, Los Angeles, California, United States of America; 5 Department of Molecular and Medical Pharmacology, University of California Los Angeles, Los Angeles, California, United States of America; 6 Eli and Edythe Broad Center of Regenerative Medicine and Stem Cell Research, University of California Los Angeles, Los Angeles, California, United States of America; Emory University, United States of America

## Abstract

Pluripotent stem cells (PSCs) hold significant promise in regenerative medicine due to their unlimited capacity for self-renewal and potential to differentiate into every cell type in the body. One major barrier to the use of PSCs is their potential risk for tumor initiation following differentiation and transplantation *in vivo*. In the current study we sought to evaluate the role of the tumor suppressor *Pten* in murine PSC neoplastic progression. Using eight functional assays that have previously been used to indicate PSC adaptation or transformation, *Pten* null embryonic stem cells (ESCs) failed to rate as significant in five of them. Instead, our data demonstrate that the loss of *Pten* causes the emergence of a small number of aggressive, teratoma-initiating embryonic carcinoma cells (ECCs) during differentiation *in vitro*, while the remaining 90–95% of differentiated cells are non-tumorigenic. Furthermore, our data show that the mechanism by which *Pten* null ECCs emerge *in vitro* and cause tumors *in vivo* is through increased survival and self-renewal, due to failed repression of the transcription factor *Nanog*.

## Introduction

Risk of tumor formation constitutes one of the major barriers to the use of pluripotent stem cell (PSC) lines in regenerative medicine. To date, only a small number of groups have focused on developing tools, or identifying molecular pathways that cause mouse or human PSC lines to undergo cultural adaptation and neoplastic progression [1], [2], [3], [4], [5], [6], [7], [8], [9]. One of the major measures of PSC neoplastic progression is the acquisition of aneuploidy identified through routine karyotyping, or by subkaryotypic changes identified by techniques such as comparative genomic hybridization [1], [4], [10]. Accompanying these molecular diagnostic tools are emerging functional *in vitro* assays for distinguishing aneuploid or adapted PSC lines from euploid parental lines. These include efficiency of re-plating from single cells, growth rate, dependence on exogenous growth factors, reduced levels of spontaneous differentiation, colony appearance, apoptosis and in some cases CD30 surface marker expression [1], [4], [5], [6], [8], [9], [10], [11]. More recently, *in vivo* assays that monitor teratoma size and numbers of failed-to-differentiate cells called embryonic carcinoma cells (ECCs) within PSC-derived teratomas have been successfully used to confirm the identity of adapted PSC lines [4], [7], [8].

Neoplastic progression of differentiated somatic cells used for cell based therapy is a critical problem [4]. However, failure to execute differentiation in a small fraction of cells that could contaminate the donor cells used for transplantation is also critical to PSC tumorigenicity, as the most common tumor type documented after transplantation of differentiated donor cells derived from PSCs are teratomas [3], [12], [13], [14], [15], [16]. In one study using murine induced pluripotent stem (iPS) cells, it was shown that the number of Nanog-positive ECCs that persisted during neurosphere differentiation *in vitro* correlated with teratoma formation of the transplanted neurospheres *in vivo* [3]. However, the mechanism by which persistent ECCs survive during differentiation is not known. In recent work, we determined that the emergence of ECCs *in vivo* from PSC-induced teratomas is associated with reduced expression of the tumor suppressor *phosphatase and tensin homologue* (*Pten)* [17]. Therefore, in the current study, our goal was to determine the consequence of *Pten* null mutations in tumorigenicity of differentiated murine embryonic stem cells (ESCs).

## Materials and Methods

### Cells

*Pten*+/+ and −/− ESC were previously published [18]. *Pten* short hair-pin (shp) knockdown cells were generated by transfecting *Pten*+/+ cells with a lentivirus containing a *Pten* specific shp construct. ESCs were maintained on mitomycin C treated fibroblast feeders in Knockout DMEM (Invitrogen) containing 15% Defined lot tested FBS (Hyclone Lot # ATJ 33070), 1 X non essential amino acids (Invitrogen), 1X Pen/Strep (Invitrogen), 1 X L-Glutamine (Invitrogen), 55 mM beta-mercaptoethanol (Invitrogen), and 1000 units/ml LIF (Chemicon). Differentiation involved plating cells in ESC media minus LIF, plus 10 µM retinoic acid (Sigma) for four days without changing the media.

### Karyotype

G-banded karyotyping was performed by Cell Line Genetics (http://www.clgenetics.com) (Madison, WI). Cells were submitted for karyotype by first culturing off feeders for one passage in T-25 flasks. Cells were shipped overnight in ESC media as live cells, and Cell Line Genetics subsequently performed metaphase spreads and Gimsea staining before counting 20 metaphases for each cell line to determine karyotype.

### Generation of teratomas

Ethics Statement: Surgery was performed following Institutional Approval for Appropriate Care and use of Laboratory animals by the UCLA Institutional Animal Care and Use Committee (Chancellor's Animal Research Committee (ARC)), Animal Welfare assurance number A3196-01. Briefly, for testicular tumors, a single incision was made in the peritoneal cavity and the testis was pulled through the incision site. Using a 27-gauge needle, 5×10^5^ ESCs, or fewer, in a volume of 50 µl 0.5X Matrigel (BD) were transplanted into the testis of adult SCID mice. Four to six weeks after surgery, mice were euthanized and the tumors removed for histology, flow cytometry and immunohistochemistry.

### Flow cytometry and cell sorting

A single cell suspension of the cell lines was generated by digestion for 5 minutes at 37°C in 5% CO_2_ in 0.25% Trypsin EDTA (Invitrogen). Single cell suspensions of tumors were generated by dissection of the tumor into 1 mm^2^ pieces followed by incubation in 1 mg/ml collagenase in high glucose DMEM for 2 hours at 37°C in 5% CO_2_. Cells were centrifuged for 5 min at 1000 rpm and resuspended in PBS with 1% BSA. For extracellular staining, SSEA1 (1∶100 DSHB) and c-kit (1∶200 BD-Pharmagen) antibodies were used for 1–2 million cells per ml of PBS/BSA. Cells were incubated with antibody for 20 minutes at 4°C, washed, incubated for 5 minutes at 4°C in PBS/BSA and washed again. Cy5 conjugated goat anti mouse IgG and IgM (1∶500) and PE conjugated goat anti rat IgG (1∶1000) (both Jackson ImmunoResearch) were used. Internal staining for Oct4 was performed using the Cytoperm/Cytofix Kit (BD). Primary Oct4 (N-19) antibody (Santa Cruz) was used at 1∶100 and FITC conjugated donkey anti goat secondary antibody (Jackson ImmunoResearch) was used at 1∶200. Cells were incubated with secondary antibody for 20 minutes, washed and resuspended in PBS/BSA for analysis on a BD Biosciences LSR II. Annexin V staining (BD Annexin V kit) was performed according to manufacturers instructions. Cells were prepared for FACS as for flow cytometry with the following exceptions. 7AAD was added to samples to exclude dead cells from sorts. For some samples MACS bead conjugated to SSEA1 antibody (Miltenyi Biotech) was added to the cells at a ratio of 20 µl antibody to 80 µl staining buffer per 1×10^7^ cells and incubated at 4°C for 20 minutes. Fluorescent secondary antibody was then added and incubated for 20 minutes at 4°C. Cells were then washed and resuspended in staining buffer. MACS separation was performed on MS columns in a MiniMACS separation unit (Miltenyi Biotech) following the manufacturer's instructions. Samples enriched for SSEA1 were then sorted on a Becton Dickenson FACS-ARIA.

### Histology and Immunofluorescence

Tumors were fixed in 4% paraformaldehyde for at least 24 hours at room temperature. Tissue was embedded in paraffin and 5 µm sections were cut for analysis. Sections were stained with hematoxylin and eosin for histology. For immunofluorescence, sections were deparafinized and re-hydrated followed by antigen retrieval in 10 mM Tris Base, 1 mM EDTA, 0.05% Tween 20 at 95°C for 40 minutes. Sections were washed in 20 mM Tris-HCl pH 7.4, 0.15 mM NaCl and 0.05% Tween 20, permeablized in 0.1% Triton X-100 in PBS and blocked in 5% normal donkey serum, 0.05% Tween in PBS. Primary antibodies against SSEA1 (1∶100 – DSHB) and Oct3/4 (1∶100 – Santa Cruz Biotech) were incubated on the sections for 16 hours at 4°C. Sections were incubated in species specific FITC or TRITC conjugated secondary antibodies (Jackson ImmunoResearch) at 1∶200 for 1 hour at room temperature. Sections were mounted in Prolong Gold Anti-fade Reagent with DAPI (Invitrogen) and photographed on an LSM 510 confocal microscope.

### Generation of secondary testicular tumors by serial transplantation

SSEA1 positive cells from primary tumors were isolated by Magnetic Activated Cell Sorting (MACS) following labeling with primary antibody against SSEA1. SSEA1+ cells were then labeled with a magnetic bead conjugated rat anti-mouse IgM secondary antibody (Miltenyi Biotech). 2.5×10^5^ SSEA1 positive cells were re-transplanted in 50 µl of 0.5X Matrigel. All experiments were completed at 6–8 weeks following transplantation.

### RT- and Semi-Quantitative Real Time PCR

RNA was extracted using the RNeasy Mini Kit (Qiagen) and reverse-transcribed with Superscript II Reverse Transcriptase (Invitrogen). For RT PCR, following reverse transcription, PCR was performed on the samples using the primers *Oct4* (F5′ AGTCTGGAGACCATGTTTCTGAAGT R5′ TACTCTTCTCGTTGGGAATACTCAATA), *Nanog* (F5′CAGAAAAACCAGTGGTTGAAGACTAG R5′ GCAATGGATGCTGGGATACTC), *Sox2* (F5′CACAACTCGGAGATCAGCAA R5′CTCCGGGAAGCGTGTACTTA) and *Gapdh* (F5′ACCACAGTCCATGCCATCAC R5′TCCACCACCCTGTTGCTGTA). For semi-quantitative Real Time PCR, PCR was performed on the cDNA according to the manufacturer's protocol using a BioRad iQ iCycler with Taqman probes for *Gapdh*, *Grb10*, *Asb4*, *Slc16a12* and *Tspan8* (ABI) or SYBR Green (Roche Applied Science) and *Gapdh*, *Oct4*, *Sox2* and *Nanog* primers.

### Western blot

Protein was extracted using M-PER cell lysis reagent (Thermo Scientific, Rockford, IL). Protein concentration was measured using the Pierce BCA Protein assay (Thermo Scientific, Rockford, IL) and 25 µg of total protein was subjected to electrophoresis on 12% NuPAGE Novex Bis-Tris gels (Invitrogen, Carlsbad, CA) according to manufacturer's instructions. Protein was transferred to Hybond ECL nitrocellulose (Amersham, Buckinghamshire, UK) at 4°C using NuPAGE transfer buffer according to manufacturer's instructions (Invitrogen). For immunoblotting, membranes were blocked in Tris buffered saline (TBS) pH 7.4 containing 0.1% Tween 20 (Sigma) (TBST) containing 5% Carnation Milk Powder and 10% Fetal bovine serum. Primary antibodies: Pten, phospho Pten, pan Akt, phospho Akt S308, phospho Akt T473, phospho Pdk1, phospho p53 S15, phospho Gsk3β S9 (all Cell Signaling), Nanog and b-actin (both Abcam) and Oct4 (Santa Cruz), were incubated with the membranes in 5% BSA or milk buffer at 4°C overnight. Membranes were washed 3x in TBST before adding secondary antibodies conjugated to peroxidase for 1 hour in milk buffer. Membranes were then washed three times in TBST before incubation in ECL Western blotting Analysis Reagent (Amersham, Buckinghamshire, UK) according to manufacturer's instructions. Membranes were placed next to Hyperfilm (Amersham, Buckinghamshire, UK) for at least 5 minutes prior to developing.

### Intracellular Lactate Assay

Cells were grown in mESC media or in differentiation media on gelatin for 4 days. 200,000 cells were analyzed for each condition in triplicate. Intracellular lactate was assayed using the BioVision Lactate Assay Kit according to manufacturers instructions. OD 570 was analyzed using a Molecular Devices SpectraMAX Plus plate reader.

### Microarray

All Microarray data is MIAME compliant and the raw data is deposited at GEO (GSE25465). Microarray targets were prepared using Nu-GEN WT-Ovation® FFPE RNA Amplification System and FL-Ovation® cDNA Biotin Module V2, and then hybridized to the Affymetrix Mouse Genome 430 2.0 Array. Data analyses were performed using Partek® Genomics Suite Version 6.4. Three biological replicates were analyzed for each cell types. Differentially expressed genes between RA treated SSEA1/c-kit positive *Pten*+/+ and −/− ESCs were selected at ≥1.5 fold and p<0.05. Bio-functional analysis was performed using Ingenuity pathways Analysis 7.6.

### siRNA knockdown

Cells were plated at 50,000 cells/well of a 6 well plate in differentiation media containing 10 µM RA. One day later, 5 µl of 10 µM ON-TARGETplus Non-targeting Pool and Nanog ON-TARGETplus SMARTpool (Thermo Scientific) siRNAs were added using 3 µl of Dharmafect 1 (Thermo Scientific) per well according to manufacturer's instructions. Cells were harvested on day 4 and analyzed as in previous experiments.

## Results

### *Pten*−/− mESC proliferate faster than wild type PSCs and generate larger teratomas

**Figure 1:**
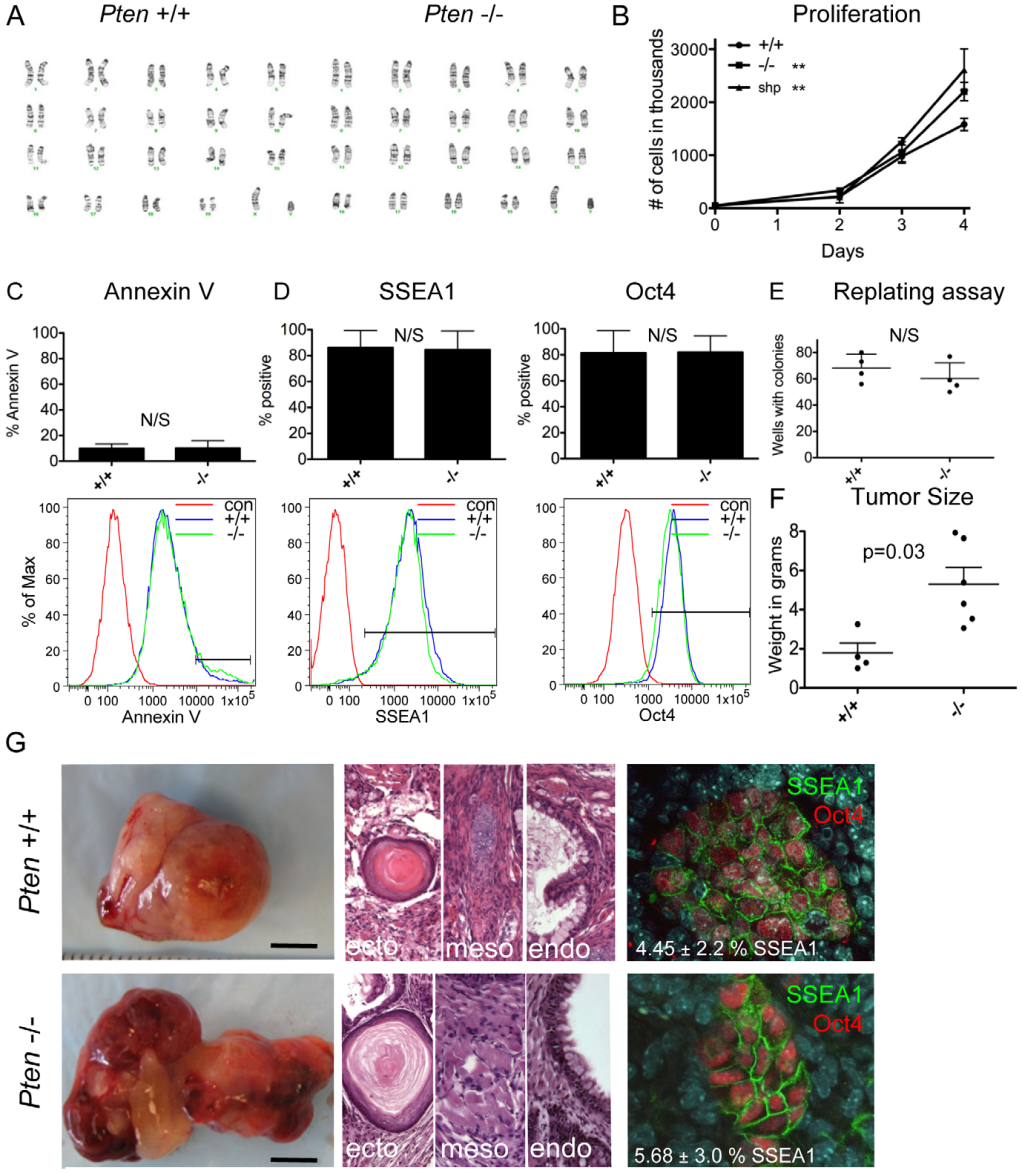
*Pten* null mutant ESCs generate larger tumors than wild type with a similar rate of *in vivo* ECC generation. (A) Karyotype of wild type and *Pten*−/− ESCs (B) Proliferation of *Pten*+/+, −/− and shp knockdown murine ESCs under self renewing conditions. (C) Annexin V staining by flow cytometry on day 4 in self-renewing culture. (D) SSEA1 and Oct4 staining by flow cytometry on day 4 of self-renewing culture. (E) Re-plating assay showing numbers of wells with alkaline phosphatase (AP) positive colonies 6 days after sorting 10 cells/well into a 96 well plates. (F) Total tumor weight after 6 weeks. *Pten*−/− tumors were significantly larger by non-parametric analysis. (G) Testicular tumor derived from wild type or *Pten*−/− ESCs (Scale bar = 0.5 cm). Histology of tumors showing derivatives of ectoderm (ecto), mesoderm (meso) and endoderm (endo) (Magnification 200×). Immunofluorescence for SSEA1 and Oct4 in tumor sections and percentage SSEA1+ by flow cytometry (mean ± SD) (Magnification 400×). **  =  p<0.005, N/S  =  not significant.

One of the most robust measures of neoplastic progression of PSCs under self-renewing conditions is faster growth rate and to some extent protection from apoptosis. Therefore, in the first set of experiments, karyotypically normal wild type and *Pten*−/− ESCs (Fig. 1A) were subjected to proliferation and apoptosis assays. We confirmed previous results [18], that *Pten*−/− and *Pten* shRNA knockdown ESCs have a faster growth rate under self-renewing conditions compared to wild type (Fig. 1B). However, this was not accompanied by decreased apoptosis using Annexin V staining (Fig. 1C). Next, we addressed whether *Pten*−/− ESCs exhibit reduced spontaneous differentiation under self-renewing conditions by evaluating SSEA1 and Oct4 expression in individual cells by flow cytometry (Fig. 1D). Our results show that *Pten*−/− ESCs are equivalent to wild type, and loss of *Pten* does not promote resistance to spontaneous differentiation in the presence of LIF. To address re-plating efficiencies, we sorted single SSEA1 positive ESCs into 96 well plates at 10 cells per well using FACS, and counted colony forming potential of the re-plated ESCs following alkaline phosphatase staining (Fig. 1E). These results show that re-plating efficiency of undifferentiated ESCs is equivalent between wild type and *Pten*−/− cells under self-renewing conditions.

In order to test teratoma initiating potential, we transplanted 5×10^5^ wild type or *Pten*−/− ESCs into testes of SCID mice (Fig. 1F–G). Quantification of total tumor burden revealed that *Pten*−/− ESC teratomas were approximately 3 fold larger than wild type at 6 weeks (Fig. 1F) (n = 6). Analysis of the tumors by histology revealed robust differentiation into all three germ layers, indicating that *Pten*−/− ESCs are capable of lineage differentiation as teratomas *in vivo* (Fig. 1G). ECC emergence in teratomas *in vivo* following transplantation of human PSCs is a hallmark of neoplastic progression [4], [7]. In contrast to human PSCs, transplantation of murine ESCs or murine egg cylinders results in teratomas that contain obvious clusters of failed-to-differentiate ECCs that can be serially transplanted [8], [17], [19]. The differences between mouse and human ESCs in this assay is not clear and may be due to host compatibility or an inherent difference between species. Therefore, given that diploid mouse ESC derived-teratomas contain ECCs, we next explored whether generation of teratomas from undifferentiated *Pten*−/− ESCs results in an increase in the percentage of ECCs in teratomas relative to wild type. Immunofluorescence of teratoma sections revealed that both wild type and *Pten*−/− tumors contained clusters of ECCs that co-stain for SSEA1 and Oct4 (Fig. 1G). Flow cytometry for SSEA1 in tumors derived from mutant and wild type ESCs revealed that the percentage of SSEA1 positive cells was not significantly different between mutant (5.68±3.0) and wild type (4.45±2.2) tumors (n = 5) (Fig. 1G). In order to evaluate teratoma-initiating potential of the ECCs derived from the primary teratomas, we sorted freshly dissected tumors and re-transplanted the SSEA1 positive cells into host SCID mice without intervening culture. Our data demonstrate that the ECCs from both genotypes are tumorigenic and can reconstitute immature teratomas that are indistinguishable from the primary tumors and from each other (Fig. S1). Taken together, using six previously reported functional assays for neoplastic progression of PSCs (proliferation rate, protection from apoptosis, protection from spontaneous differentiation, increased colony forming potential, increased teratoma size and increased proportion of ECCs in teratomas), only proliferation rate and increased teratoma size were found to associate with ESCs containing a null mutation in *Pten*.

### *Pten*−/− cells are capable of generating teratomas post-differentiation

**Figure 2:**
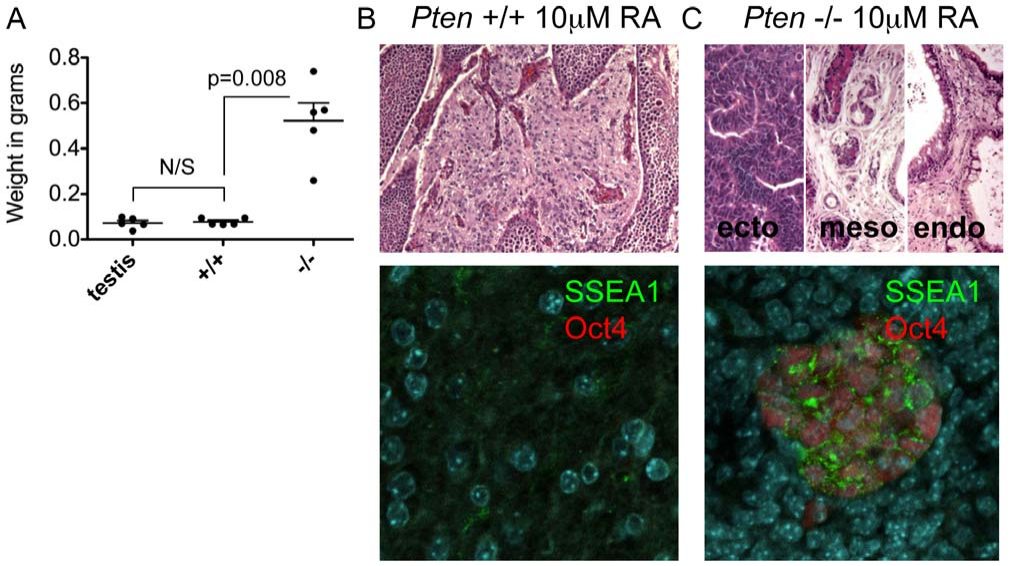
Differentiation of *Pten* null mutant ESCs does not abolish tumor formation. (A) Tumor burden of differentiated wild type and *Pten*−/− ESCs. *Pten*−/− tumors were significantly larger by non-parametric analysis. (B) Non-invasive plug of cells identified after transplanting differentiated wild type cells (Magnification 200×). Immunofluorescence of plug showing no residual undifferentiated cells (Magnification 400×). (C) Histology of teratocarcinomas generated after transplanting differentiated *Pten*−/− ESCs (Magnification 200×). Immunofluorescence showing clusters of failed to differentiate SSEA1/Oct4 positive cells in tumors (Magnification 400×).

Given that differentiation is the first step in using PSCs for regenerative medicine, we assayed teratoma-initiating potential of wild type and *Pten*−/− ESCs following differentiation *in vitro*. Differentiation was conducted for four days in the presence of 10 µM retinoic acid (RA) without LIF. No difference was seen in the morphology of differentiating *Pten* wt and −/− ESCs (Fig. S2). To determine if there was a difference in teratoma formation ability of wild type and *Pten*−/− differentiated cells, 5×10^5^ differentiated cells were transplanted into SCID mice. Visual inspection and quantification of testicles transplanted with differentiated cells indicated that tumors only developed in testicles transplanted with differentiated *Pten*−/− ESCs and not wild type ESCs. Testicles transplanted with *Pten*−/− differentiated cells were five times larger than unmanipulated testicles or testicles transplanted with differentiated wild-type cells of equal cell number (Fig. 2A). By histology we show that testicles transplanted with differentiated wild type cells contained no evidence of tumor formation, however in 1/4 samples (shown), we identified a plug of fibroblast-like cells that were non-invasive and did not stain for pluripotency markers (Fig. 2B). In contrast, transplantation of differentiated *Pten*−/− cells resulted in invasive immature teratomas that destroyed most of the testicular tissue. These teratomas contained a small SSEA1+/Oct4+ ECC component that was serially transplantable in 9/9 transplants (Fig. 2C).

**Figure 3:**
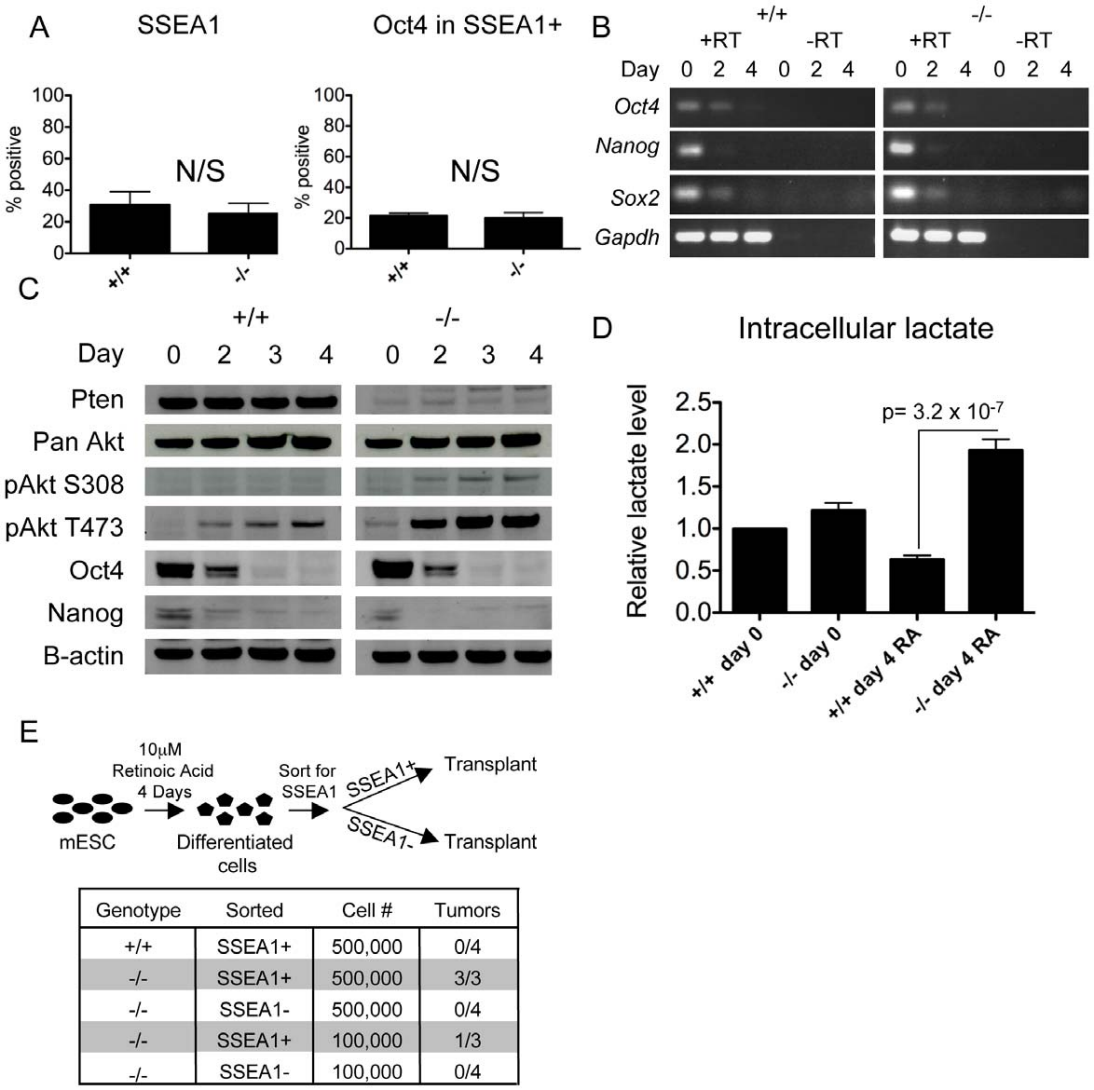
*Pten*−/− cells are able to down-regulate pluripotency genes normally upon differentiation and the tumorigenic population of differentiated cells is confined to the SSEA1 positive cells. (A) Flow cytometry to evaluate percentage of SSEA1 and Oct4/SSEA1 double positive cells in the differentiated population on day 4 of differentiation. (B) RT-PCR on days 0, 2 and 4 of differentiation for *Oct4, Nanog, Sox2* and *Gapdh*. (C) Western blot comparing wild type and *Pten*−/− cells at days 0, 2, 3 and 4 days of differentiation in the presence of retinoic acid (RA). (D) Intracellular lactate in undifferentiated and RA differentiated wild type and *Pten*−/− ESCs normalized to intracellular lactate on day 0 in wild type. (E) Diagram of procedure used and table of tumor formation from SSEA1+ or – cells from *Pten*+/+ and −/− differentiated cultures. N/S  =  not significant.

In order to determine whether the teratoma-initiating potential in the differentiated population was due to an increased number of failed to differentiate cells *in vitro* as shown for transformed iPS cells [3], we performed flow cytometry for Oct4 and SSEA1 on day 4 of differentiation, and found no statistically significant difference in the percentage of SSEA1 positive cells or SSEA1/Oct4 double positive cells compared to wild type (Fig. 3A). Furthermore, RT-PCR at days 2 and 4 of differentiation in wild type and *Pten*−/− cells suggested that repression of pluripotent transcription factor *Oct4, Nanog* and *Sox2* RNA at day 4 of differentiation was similar between the two genotypes indicating that the *Pten*−/− cells do not have an intrinsic defect in the ability to repress pluripotent transcription factor RNA levels in the majority of differentiating cells (Fig. 3B). Next we examined protein levels and phosphorylation status of members of the PI3k/Akt pathway as well as Oct4 and Nanog during *in vitro* differentiation. The activating post-translational phosphorylation of Serine 308 and Threonine 473 on Akt were increased in *Pten*−/− differentiated cells at days 2, 3 and 4 of differentiation relative to wild type (Fig. 3C). However, repression of Nanog and Oct4 protein in the general population during differentiation was not overtly affected by loss of *Pten* (Figure 3C), in agreement with the RT-PCR data (Figure 3B).

It has been proposed that the high glycolytic activity in cancer cells is due to increased activity of Akt [20] and it has been shown that embryonic stem cells have high glycolytic activity in the undifferentiated (tumorigenic) state and lower glycolytic activity in the differentiated (non-tumorigenic) state [21], [22]. To examine if metabolism is affected in the absence of *Pten*, we performed an intracellular lactate assay and found that *Pten*−/− differentiated cells have significantly higher levels of lactate relative to wild type (Figure 3D).

### The tumorigenic differentiated *Pten*−/− cells are confined to a small population

To determine if tumorigenicity can be assigned to a subpopulation of differentiated cells, we transplanted the SSEA1 positive and negative fractions separately into SCID mice (Figure 3E). Initially we compared sorted SSEA1 positive cells from wild type and *Pten*−/− differentiated cultures at 500,000 cells per transplant, and determined that teratoma inducing ability was exclusively found in differentiated SSEA1 positive cells that lacked *Pten* and not wild type. Next we compared tumor-initiating potential between SSEA1 positive and negative cells following differentiation of *Pten*−/− ESCs for four days in RA. Our results show teratoma-initiating ECC potential is not equal between the sorted populations, and instead ECCs are found exclusively in the SSEA1 positive fraction of *Pten*−/− differentiated cells with SSEA1 negative cell fractions not forming tumors at all (Fig. 3E).

**Figure 4:**
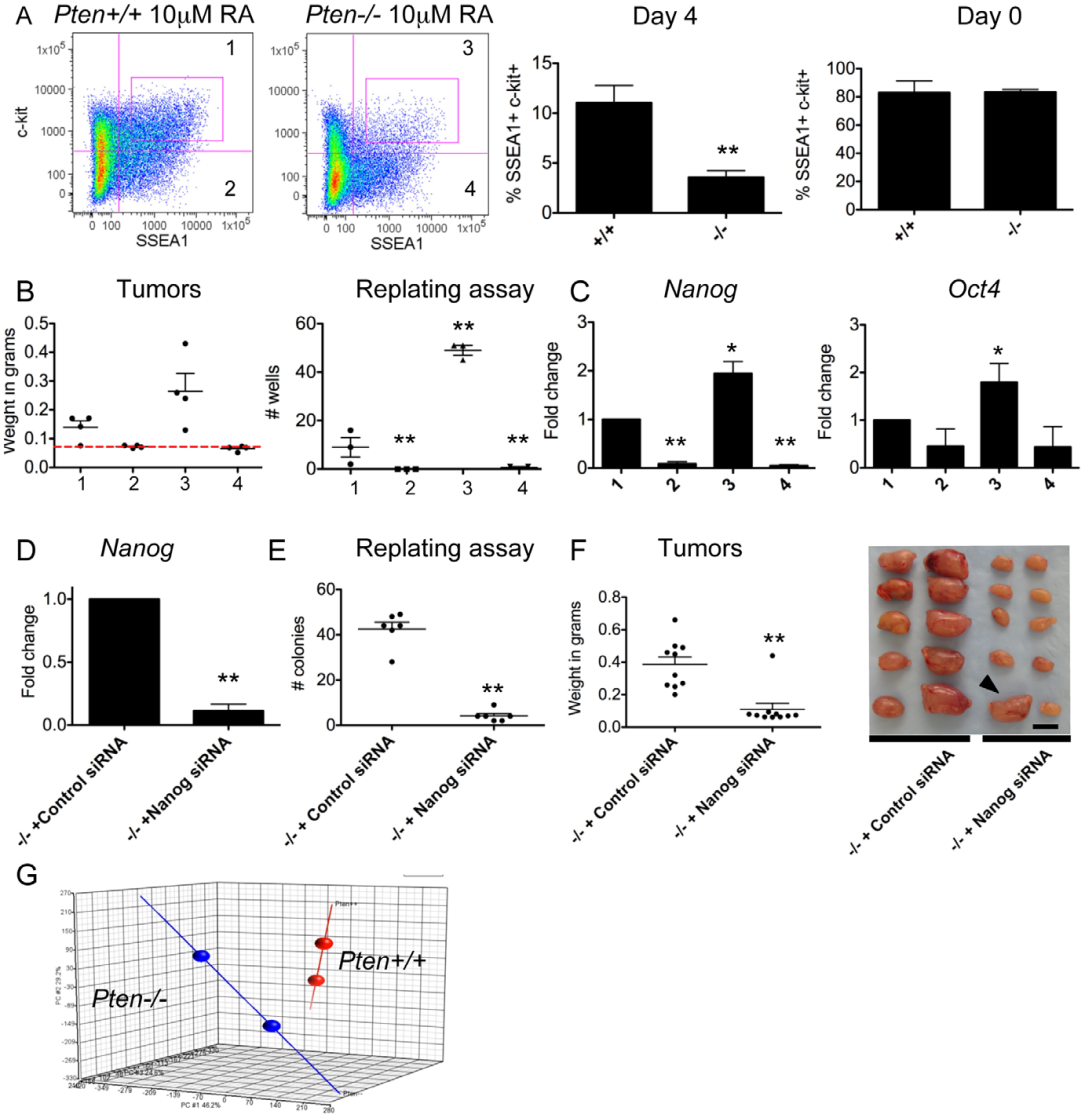
The SSEA1/c-kit double positive subpopulation of *Pten*−/− differentiated cells is responsible for tumorigenicity. (A) Flow plots showing SSEA1 and c-kit expression of wild type and *Pten*−/− cells on day 4 of differentiation in 10 µM RA. Percentage of SSEA1/c-kit double positive cells on day 4 of differentiation and undifferentiated (Day 0) cultures. (B) Tumor weight of 10,000 transplanted cells and graph indicating number of wells containing alkaline phosphatase positive colonies 6 days after sorting 100 cells into each well of a 96 well plate. 4 fractionated populations (1–4) used as indicated in flow plots in A. (C) Semi-quantitative real time PCR for *Nanog* and *Oct4* in the fractionated populations as indicated in panel A. (D) Semi-quantitative real time PCR for *Nanog* in *Pten*−/− cells on day 4 of differentiation following addition of a control or *Nanog* specific siRNA pool. (E) Re-plating assay showing number of AP positive colonies using 5000 SSEA1/ckit positive cells/well of a 6 well plate from day 4 differentiated *Pten*−/− cells with control or *Nanog* specific siRNAs. (F) Tumor weight and images of testes transplanted with 500,000 differentiated *Pten*−/− cells with control or *Nanog* specific siRNAs. Arrowhead indicates tumor generated from *Nanog* siRNA treated cells. (G) PCA map of microarray on SSEA1+/c-kit+ wild type and *Pten*−/− differentiated cells. *  =  p<0.05 **  =  p<0.005, N/S  =  not significant.

While the teratoma-initiating potential was confined to the SSEA1 positive fraction, teratoma-initiating ability was attenuated when using 100,000 cells (Fig. 3E). This suggests that not every SSEA1 positive cell is tumorigenic. Therefore, in order to more specifically define the ECC population within the SSEA1 fraction, we examined expression of the surface marker c-kit, as this receptor is also expressed on carcinoma *in situ*, the precursors of testicular germ cell tumors called teratocarcinomas, the malignant counterpart of teratomas [23]. Using flow cytometry we identified three times as many SSEA1/c-kit double positive cells in the wild type samples relative to *Pten*−/− at day 4 of differentiation (Fig. 4A). Paradoxically, this is opposite to what would have been predicted for a cell population that functionally has increased capacity for inducing teratomas [3]. This effect was specific to differentiated cells as no difference was observed in the percentage of SSEA1/c-kit double positive cells when cultured in the self-renewing undifferentiated state (Day 0) (Fig. 4A). To examine the tumorigenic potential of the SSEA1/c-kit double positive cells, we sorted and transplanted 10,000 SSEA1/c-kit double positive (sample 1 and 3), or SSEA1 positive c-kit negative cells (samples 2 and 4). Teratoma-initiating potential was enriched in the SSEA1/c-kit double positive population and never observed in SSEA1+ cells negative for c-kit (Fig. 4B). Furthermore, teratomas derived from *Pten* −/− SSEA1/c-kit double positive cells were larger than wild type, and were metastatic indicating that despite the lower numbers of SSEA1/c-kit double positive cells that emerged following *in vitro* differentiation of *Pten*−/− cells, the tumorigenic potential of this double positive population is significantly greater than wild type. In order to generate a rapid *in vitro* assay for recapitulating the increased teratoma-inducing potential of SSEA1/c-kit *Pten*−/− differentiated cells, we designed a re-plating assay in which ESCs were differentiated in the presence of RA for four days and 100 SSEA1/c-kit double positive cells/well were plated as single cells into 96 well plates. Colony forming ability was assessed 6 days after re-plating by alkaline phosphatase staining (Fig. 4B). Using this second assay, we clearly show that *Pten*−/− differentiated teratoma-initiating ECCs have 5 times greater colony forming potential than wild type cells, suggesting increased capacity for ECC survival and self renewal over wild type (Fig. 4B).

In order to determine whether pluripotent gene expression was altered in the SSEA1/c-kit double positive *Pten*−/− differentiated cells relative to wild type, we examined the expression of *Oct4*, *Nanog* and *Sox2* by semi-quantitative real time PCR (Fig. 4C and data not shown). Our results indicate that unlike the majority of differentiated cells, which repress these transcription factors (Fig. 3B), *Nanog* and *Oct4* mRNA exhibited a modest yet statistically significant increase in differentiated *Pten*−/− SSEA1/c-kit double positive cells (sample 3) relative to differentiated SSEA1/c-kit wild type sorted cells (sample 1) (Fig. 4C). *Sox2* showed no statistically significant change relative to wild type (data not shown). Interestingly, we found that expression of *Nanog* was significantly higher in the tumorigenic SSEA1/c-kit double positive cells (sample 3) compared to the non-tumorigenic SSEA1 positive c-kit depleted cells (sample 4), suggesting that expression of *Nanog* specifically associates with tumorigenicity in this model. To test the role of *Nanog* in the tumorigenicity of the *Pten*−/− ECCs, we knocked down *Nanog* during differentiation of *Pten*−/− cells in the presence of RA and evaluated colony forming potential of the emerging ECCs. Using *Nanog* specific siRNAs we were able to knockdown *Nanog* RNA by 93% relative to scrambled siRNA controls on day 4 of differentiation (Fig. 4D). To test whether *Nanog* repression by siRNAs attenuated ECC emergence during differentiation, we re-plated 5000 SSEA1/c-kit double positive *Pten*−/− differentiated cells in 6-well plates, and identified a significant 10-fold reduction in the numbers of alkaline phosphatase positive colonies derived from the *Pten*−/− *Nanog* siRNA treated differentiated cultures verses the control *Pten*−/− differentiated cultures (Fig. 4E). To determine if the knockdown of *Nanog* during differentiation would reduce the tumorigenic potential of the *Pten*−/−cells *in vivo*, we transplanted 500,000 *Pten*−/− cells differentiated for 4 days with either control or *Nanog* siRNAs. Teratomas formed in 10 of 10 transplants with the control siRNAs (as expected from Fig. 2A and C). In contrast, only 1 of 10 transplants resulted in a tumor following treatment with *Nanog* specific siRNAs (Fig. 4F). Thus we were able to significantly reduce the tumorigenicity of *Pten*−/− cells by differentiating in the presence of siRNAs against *Nanog*.

Finally, in order to identify additional downstream effectors in the *Pten*−/− ECCs that emerge with differentiation, we performed microarray analysis comparing wild type and mutant SSEA1/c-kit double positive sorted cells after 4 days of differentiation in RA (Fig. 4G). Using principle component analysis (PCA) of the replicate samples of each genotype we found that the wild type and mutant samples clustered separately, with 85 unique genes distinguishing wild type and mutant SSEA1/c-kit sorted cells on day 4 of differentiation (Tables S1 and S2). The microarray was confirmed by semi-quantitative real-time PCR (Figure S3). Gene ontology analysis of these 85 genes identified functional groups again associated with cholesterol metabolism, response to nutrient levels and endogenous stimuli, regulation of cell growth, pyruvate metabolic processes and stem cell maintenance and development.

## Discussion

In the current study we evaluated ESCs with a null mutation in *Pten* in eight functional assays that have previously been used to distinguish culture adapted or transformed human and mouse PSCs from wild type (Table S3). The majority of these assays are conducted under self-renewing (undifferentiated) conditions, with only three assays evaluating tumorigenicity of differentiated cells. Our data demonstrate that *Pten*−/− ESCs exhibit hallmarks of neoplastic progression in just 3/8 assays when compared to wild type diploid ESCs. Furthermore, using differentiation and evaluation of failed-to-differentiate cells, the *Pten*−/− differentiated ESC samples paradoxically contain fewer tumor-initiating SSEA1/c-kit double positive ECCs relative to wild type cells *in vitro*. Transcriptionally, we show that the *Pten*−/− ECCs have modest yet significantly higher expression levels of *Nanog* and *Oct4* mRNA, and functionally show that *Pten*−/− ECCs have greater capacity for survival and self renewal in tumor and colony forming replating assays. Critically, we find that the emergence of ECCs in the *Pten*−/− cells during differentiation *in vitro* can be significantly attenuated by differenting in the presence of *Nanog* siRNAs. Together, our data demonstrate that it is not necessarily the number of failed-to-differentiate cells that contribute to increased tumorigenicity following differentiation, but also the aggressive behavior of these ECCs following transplantation, and this effect can be modulated in *Pten* mutants by actively forcing repression of *Nanog* during differentiation *in vitro*.

The role of the PI3k pathway in regulating *Nanog* and *Oct4* expression is well known [24], [25], [26]. In particular, culture of murine ESCs for 8 days in the absence of LIF, and presence of myristoylated-Akt (active form of Akt), results in the maintenance of *Oct4* and *Nanog* mRNA in the differentiating population and an increase in the percentage of ESC-like colonies. However, the proportion of undifferentiated colonies in the presence of active Akt is lower when compared to culture in the presence of LIF alone, suggesting that not every differentiated cell is capable of retaining stem cell like characteristics when exposed to active Akt in the absence of LIF [24]. Our data extend these findings to show that under conditions of differentiation in the absence of LIF and addition of RA, loss of *Pten* and increased phosphorylation of Akt attenuate the repression of *Nanog* and *Oct4* mRNA as previously reported, but only in the emerging ECC population and not in the general differentiated population. In a different study that evaluated self-renewing ESCs in the presence of LIF, blocking the PI3k pathway in murine ESCs caused repression of *Nanog* mRNA and protein [25], [26]. In agreement with this work but under differentiating conditions in the presence of RA, we showed that addition of *Nanog* siRNAs to *Pten*−/− differentiating ESCs significantly attenuated the emergence of ECCs from differentiated cultures *in vitro* and the tumorigenicity of these cells *in vivo*, therefore illustrating the importance of repressing *Nanog* downstream of the PI3k pathway to block ECC emergence during ESC differentiation.

A novel aspect of our study was the finding that only a small proportion of the *Pten*−/− differentiating cells are sensitive to retention of a stem cell-like state and retain elevated levels of *Nanog*, while the majority of *Pten*−/− cells initiate differentiation, lose tumorigenicity and repress *Nanog*. One hypothesis for this phenomenon is that the emerging ECCs identified *in vitro* are transformed germ cells which also express SSEA1 and c-kit. Support for this hypothesis is provided by the strong evidence that spontaneous differentiation of ESCs *in vitro* consistently generates a small population of germ cells within 3–5 days of culture [27], [28], [29], [30], [31], [32], together with the result that conditional loss of *Pten* in fetal germ cells invariably causes teratomas from the abnormal germ cells *in vivo* [33].

In the current study, microarray analysis of sorted SSEA1/c-kit double positive population after differentiation revealed an increase in pathways associated with glucose and cholesterol metabolism. Furthermore, we show that *Pten*−/− differentiated cells have significantly higher levels of lactate than wild type. It is known that activation of Akt increases intracellular ATP levels and accelerates both glycolytic and oxidative metabolism (for review see [20]). ESCs cultured under self- renewing conditions proliferate quickly, yet appear to meet the majority of their energy requirements by glycolysis similar to classic Warburg cancer cells [22]. The increased intracellular lactate in differentiated *Pten*−/− cells relative to wild type suggests that these cells have failed to effectively switch from glycolysis to oxidative phosphorylation. The importance of transitioning from aerobic glycolysis to oxidative phosphorylation during ESC differentiation remains to be fully explored, and the role of Akt signaling in this process warrants further investigation in light of its association with increased tumorigenicity as described here.

A role for cholesterol in ESC self-renewal and differentiation is now beginning to emerge. Previous studies have shown that inhibiting cholesterol biogenesis with statins inhibits self-renewal of murine ESCs as well as genetically abnormal human ESCs, while not affecting euploid hESC lines [34], [35]. The importance of the cholesterol pathway in mESC self-renewal has been associated with the generation of intermediates required for post-translational modification of RhoA and the Rock pathway [34]. This is a critical link as Rock inhibitors have been shown to improve survival and single cell cloning of hESCs under self renewing conditions [36], [37], [38]. Together, this data suggests that cholesterol metabolism and may also be critical for promoting increased survival and growth of *Pten*−/− ECCs under differentiating conditions, and this warrants further investigation.

Our data further illuminate the hypothesis first suggested by Harrison *et. al*. that ‘culture adaptation (and therefore neoplastic progression) can occur through differing routes’ [11]. Together this would suggest that multiple assays must be integrated in order to predict neoplastic progression of PSC lines before a given line can be used in regenerative medicine. In the current study we have shown that deletion of a specific tumor suppressor, *Pten* resulted in tumorigenicity specifically by promoting the emergence of a small number of highly tumorigenic ECC cells *in vitro,* while the remaining differentiated cells were non tumorigenic. Previously reported pathways and mechanisms of neoplastic progression have included failed senescence of differentiated transplanted cells [4], increased survival of differentiated cells [8], increased numbers of failed to differentiated cells [3], and aneuploidy [7]. The various mechanisms behind each of these routes is equally critical to understand in order to prevent PSCs that have undergone neoplastic progression from being included in clinical use.

Taken together, the use of differentiation as the starting point to measure neoplastic progression of PSC lines provides a novel method for evaluating PSC tumorigenicity. In the current study we discovered that a null mutation in a single gene, *Pten*, caused teratomas from differentiated PSCs by promoting the survival of a small population of highly tumorigenic ECC cells while the rest of the population was non tumorigenic. The survival of these ECCs is associated with failed repression of *Nanog* as well as a propensity for increased glucose and cholesterol metabolism. In conclusion, the identification of specific signaling pathways that contribute to neoplastic progression of PSCs such as the one described here, are vital as clinical applications for PSCs are currently at-hand.

## Supporting Information

Figure S1 Secondary tumors derived from SSEA1 positive ECCs from wild type and *Pten−/−* teratomas (Scale bar = 0.5 cm). Histology of tumors showing derivatives of ectoderm (ecto), mesoderm (meso) and endoderm (endo) (200× magnification). Immunofluorescence for SSEA1 and Oct4 in wild type and *Pten−/−* secondary tumor (400× magnification).
(PDF)

Figure S2 Differentiation of wild type and *Pten−/−* mESCs on days 2 and 4 in 10 µM Retinoic Acid. 100X Magnification.
(PDF)

Figure S3 RT-PCR results to confirm significantly altered genes identified by the SSEA1+c-kit+ microarray.
(PDF)

Table S1 Genes upregulated in SSEA1/c-kit *Pten−/−* mESC after 4 days of differentiation.
(PDF)

Table S2 Genes downregulated in SSEA1/c-kit *Pten*−/− mESC after 4 days of differentiation.
(PDF)

Table S3 Assays used to evaluate neoplastic progression in pluripotent stem cells and their derivatives.
(PDF)
