# Supplementary material for: Loss of Pten Causes Tumor Initiation Following Differentiation of Murine Pluripotent Stem Cells Due to Failed Repression of Nanog
Source: PLoS One. 2011 Jan 27;6(1):e16478. doi: 10.1371/journal.pone.0016478 (PMC3029365; doi:10.1371/journal.pone.0016478)
Supplement: Figure S1 — Secondary tumors derived from SSEA1 positive ECCs from wild type and Pten−/− teratomas (Scale bar = 0.5 cm). Histology of tumors showing derivatives of ectoderm (ecto), mesoderm (meso) and endoderm (endo) (200× magnification). Immunofluorescence for SSEA1 and Oct4 in wild type and Pten−/− secondary tumor (400× magnification). (PDF) [file pone.0016478.s001.pdf]

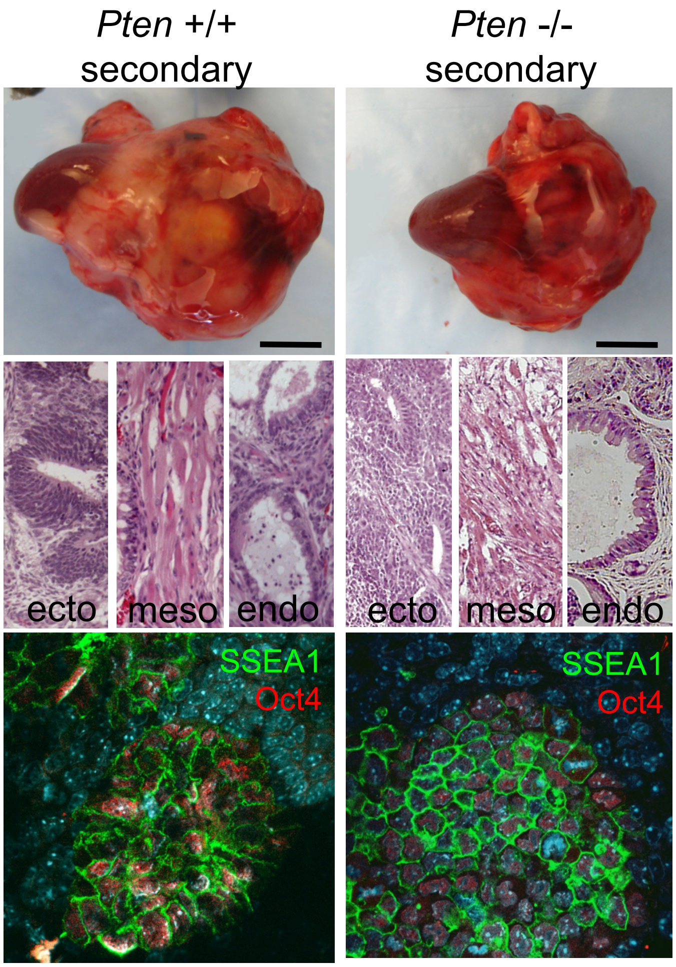

Figure S1. Secondary tumor derived from SSEA1 positive ECCs from wild type and *Pten-/-* teratomas (Scale bar = 0.5 cm). Histology of tumors showing derivatives of ectoderm (ecto), mesoderm (meso) and endoderm (endo) (200X magnification). Immunofluorescence for SSEA1 and Oct4 in wild type and *Pten*-/- secondary tumor (400x magnification).
