# Supplementary material for: Loss of Pten Causes Tumor Initiation Following Differentiation of Murine Pluripotent Stem Cells Due to Failed Repression of Nanog
Source: PLoS One. 2011 Jan 27;6(1):e16478. doi: 10.1371/journal.pone.0016478 (PMC3029365; doi:10.1371/journal.pone.0016478)
Supplement: Figure S2 — Differentiation of wild type and Pten−/− mESCs on days 2 and 4 in 10 µM Retinoic Acid. 100X Magnification. (PDF) [file pone.0016478.s002.pdf]

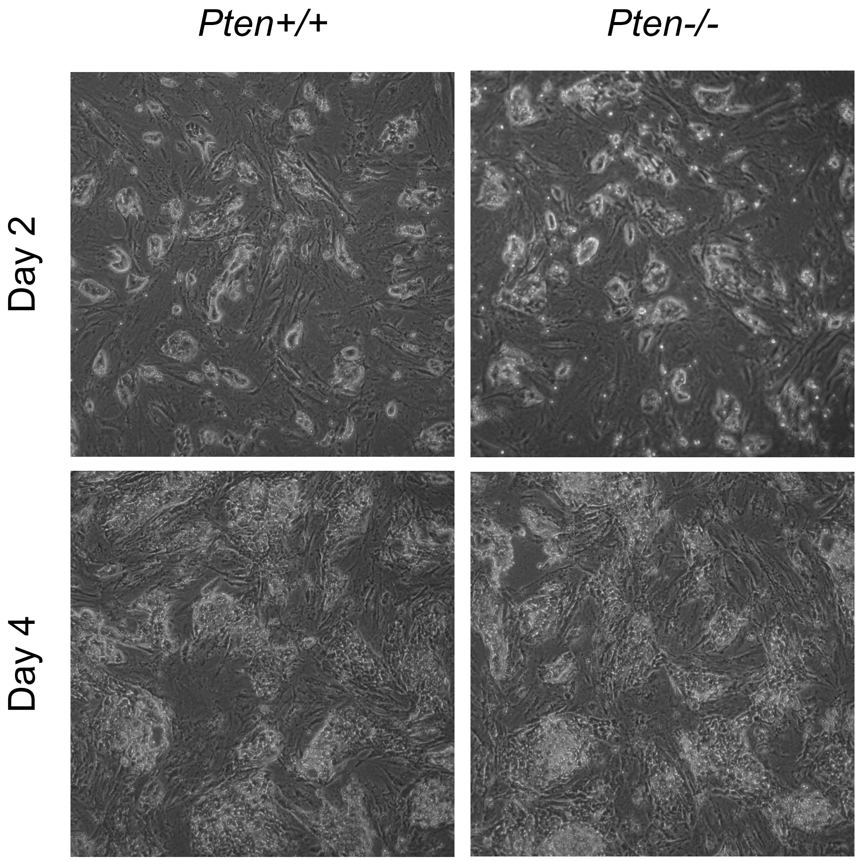

Figure S2. Differentiation of wild type and *Pten-/-* mESCs on days 2 and 4 in 10μM Retinoic Acid. 100X Magnification.
