# Supplementary material for: Loss of Pten Causes Tumor Initiation Following Differentiation of Murine Pluripotent Stem Cells Due to Failed Repression of Nanog
Source: PLoS One. 2011 Jan 27;6(1):e16478. doi: 10.1371/journal.pone.0016478 (PMC3029365; doi:10.1371/journal.pone.0016478)
Supplement: Figure S3 — RT-PCR results to confirm significantly altered genes identified by the SSEA1+c-kit+ microarray. (PDF) [file pone.0016478.s003.pdf]

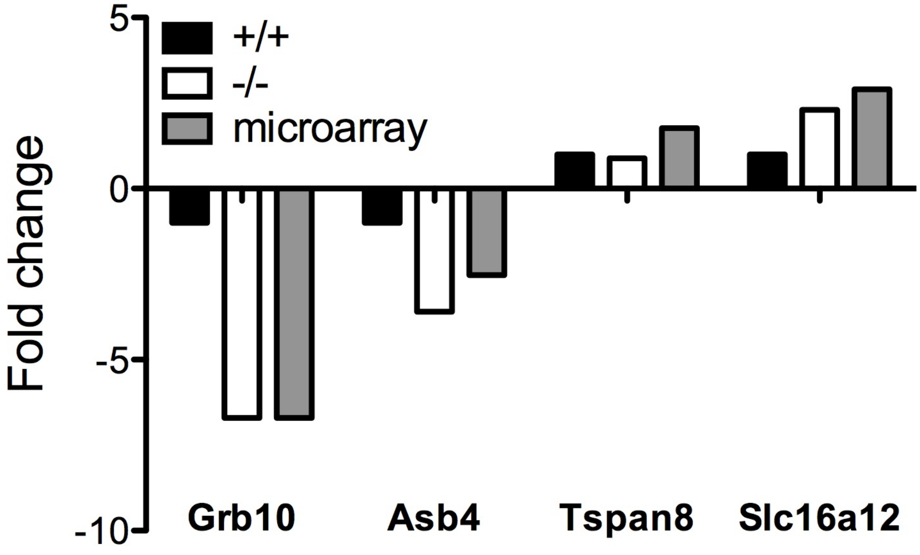

Figure S3. RT-PCR results to confirm significantly altered genes identified by SSEA1+c-kit+ microarray.
