# Supplementary material for: Loss of Pten Causes Tumor Initiation Following Differentiation of Murine Pluripotent Stem Cells Due to Failed Repression of Nanog
Source: PLoS One. 2011 Jan 27;6(1):e16478. doi: 10.1371/journal.pone.0016478 (PMC3029365; doi:10.1371/journal.pone.0016478)
Supplement: Table S1 — Genes upregulated in SSEA1/c-kit Pten−/− mESC after 4 days of differentiation. (PDF) [file pone.0016478.s004.pdf]

Table S1: Genes upregulated in SSEA1/c-kit *Pten*-/- mESC after 4 days of differentiation

Gene	Fold change
Cbx7	6.8883
Slc16a12	2.98771
Lipg	2.73076
Smyd1	2.62361
Necab1	2.57953
Crygb	2.31699
Nr0b2	2.24367
Cfh	2.14473
Tmem120a	2.1224
Ttr	2.08936
Dpys	2.0493
Prrt3	2.01719
Trib3	1.98962
Pou5f1	1.97632
Igfbp5	1.93862
Atp1a1	1.88211
Gjb3	1.86197
Gpr123	1.85893
Fgf4	1.85496
Sgce	1.83943
Abhd5	1.82365
Tspan8	1.77142
Aqp3	1.75129
Tbx4	1.73159
Pcsk9	1.72075
Coro2b	1.71921
Nupr1	1.69922
Mnx1	1.69126
Pla2g2e	1.68636
Fam167a	1.66313
Hapln1	1.63194
Ociad2	1.62471
Clps	1.61949
C130026L21Rik	1.61451
Fam129a	1.60709
Cap2	1.58966
Stra6	1.58726
Fdps	1.58647
Fbp2	1.58402
Slc30a10	1.58315
Ceacam1	1.57666
Igfbp4	1.56635
Mypn	1.56387
Nnat	1.55805
Sep4	1.55325

Lmnb1	1.5517
Vwf	1.5455
Adh1	1.5317
AI661453	1.53144
Casq1	1.51847
Ldlr	1.51537
Limk1	1.5133
Iqcg	1.51046
Mcm5	1.50513
Foxh1	1.5028
