# Supplementary material for: Loss of Pten Causes Tumor Initiation Following Differentiation of Murine Pluripotent Stem Cells Due to Failed Repression of Nanog
Source: PLoS One. 2011 Jan 27;6(1):e16478. doi: 10.1371/journal.pone.0016478 (PMC3029365; doi:10.1371/journal.pone.0016478)
Supplement: Table S2 — Genes downregulated in SSEA1/c-kit Pten−/− mESC after 4 days of differentiation. (PDF) [file pone.0016478.s005.pdf]

Table S2: Genes downregulated in SSEA1/c-kit *Pten*-/- mESC after 4 days of differentiation

Gene	Fold change (Pten-- vs. Pten++)
Grb10	-6.733825352
H19	-3.331811806
Asb4	-2.533364409
Zfp182	-2.336923627
Spock3	-2.193930273
Nts	-2.020471416
Krt6a	-1.982632142
Igf2r	-1.856462066
Csn3	-1.799953201
Pdk4	-1.782543904
H13	-1.705820943
Trh	-1.696396007
Usp26	-1.696332698
Ttll7	-1.696004722
Egr2	-1.669948115
Trpc5	-1.661187517
Gm364	-1.658157555
Flt4	-1.636152424
Zfp771	-1.618932444
Kcnip4	-1.601045162
Gm8126	-1.575512751
C79818	-1.570746419
Tpbg	-1.557195802
Wdr35	-1.550839625
Lce1f	-1.547915731
Tnc	-1.544518427
Hoxc5	-1.541675339
Pisd-ps3	-1.513113397
Klk6	-1.507256687
