# Supplementary material for: Loss of Pten Causes Tumor Initiation Following Differentiation of Murine Pluripotent Stem Cells Due to Failed Repression of Nanog
Source: PLoS One. 2011 Jan 27;6(1):e16478. doi: 10.1371/journal.pone.0016478 (PMC3029365; doi:10.1371/journal.pone.0016478)
Supplement: Table S3 — Assays used to evaluate neoplastic progression in pluripotent stem cells and their derivatives. (PDF) [file pone.0016478.s006.pdf]

Table S3: Assays used to evaluate neoplastic progression in pluripotent stem cells and their derivatives

Assay	Cell state under analysis	Genetic lesion or cell line	Reference	Pten null
Increased proliferation rate	Undifferentiated	- amp20q11.1-11.2*<br>- Mosaic+12<br>- dup(1)(q21.1q32.1)<br>- t(1;6)(p22;q15)<br>- +1 der(6)t(6;17)(q27;q1)<br>-dup (1p32–1p36)	4, 6, 9 - 11	Yes
Decreased apoptosis	Undifferentiated	- dup(1)(q21.1q32.1)<br>- dup (1p32–1p36)	5, 6, 9	No
Decreased spontaneous differentiation	Undifferentiated	- amp20q11.1-11.2*<br>- +1,der(6)t(6;17)(q27;q1)	4, 10	No
Increased colony forming capacity (cloning efficiency)	Undifferentiated	+1 der(6)t(6;17)(q27;q1)	10, 11	No
Increased teratoma size	Undifferentiated	- amp20q11.1-11.2*<br>- dup(17)t(17;X)	4, 7	Yes
Increased numbers of ECCs in teratomas	Undifferentiated	- 20q11.1-11.2*<br>- MEF-iPS<br>- MOSAIC+12<br>- dup(1)(q21.1q32.1)<br>- t(1;6)(p22;q15)	3, 4, 9	No
Re-transplant of tumor ECCs	Teratoma stem cells	- dup(17)t(17;X)<br>- multiple	7, 17	Yes
Increased numbers of failed to differentiate cells in vitro	Differentiated	- amp20q11.1-11.2*<br>- MEF-iPs	3, 4	No
Increased re-plating efficiency of failed to differentiate cells	Differentiated	Pten null mutation	This study	Yes

(amp = amplification); * = detected by array CGH (subkaryotypic lesion); dup = duplication; t= translocation.
